# Concentrations of H1-Receptor Antagonist in the Human Nasal Mucosa

**DOI:** 10.1155/2009/495186

**Published:** 2009-11-25

**Authors:** Kenji Takasaki, Kaori Enatsu, Hidetaka Kumagami, Haruo Takahashi

**Affiliations:** Department of Otolaryngology, Head and Neck Surgery, Nagasaki University Graduate School of Biomedical Sciences, 1-7-1 Sakamoto, Nagasaki 852-8501, Japan

## Abstract

*Aims*. To measure blood and tissue concentrations of the H1-receptor antagonist, bepotastine besilate (BB). *Methods*. Participants included 14 men and six women, whose age ranged from 13 to 76 years, with chronic rhinosinusitis, who underwent endoscopic sinus surgery at our university hospital. Among them, 10 participants had allergic rhinitis (AR) (Group I), and others did not have AR (Group II). Nasal mucosa and blood were collected 55 to 130 minutes after oral administration of BB 10 mg. Concentrations of the agent in the serum and nasal mucosa were measured by high-performance liquid chromatography. *Results*. Concentrations of BB of the serum in Group I and II were 98 ± 32 ng/mL and 112 ± 39 ng/mL. Those of the nasal mucosa tissue in Groups I and II were 101 ± 36 ng/g and 132 ± 44 ng/g. There was no significant difference in the values of concentration of BB between the serum and the nasal mucosa in either Group I or II (*P* = .757 and *P* = .2662, resp., Paired *t*-test). *Conclusion*. This preliminary study is considered the first report on the concentration of H1-receptor antagonists in nasal mucosa. The prompt absorption and transition to the nasal mucosa of BB seems to have an effect on allergic rhinitis.

## 1. Introduction

The H1-receptor antagonist, bepotastine besilate, was developed in Japan and is now widely used for allergic rhinitis in Japan and Korea [1, 2]. Measurement of the concentrations of the H1-receptor antagonist in nasal mucosa may provide more direct information about the correlation between the local concentration of the agent and nasal histamine blocking action. However, to our knowledge, there has never been a report measuring the concentrations of any H1-receptor antagonist in nasal mucosa. The aim of this study is to measure blood and tissue concentrations of bepotastine besilate in patients with chronic rhinosinusitis (CRS).

## 2. Subjects and Methods

Participants included 14 men and six women, whose age ranged from 13 to 76 years (mean age of 48), with CRS, who underwent endoscopic sinus surgery at our university hospital. Their CRS was diagnosed by their clinical symptoms and signs (nasal discharge, nasal obstruction, and so on). Computed tomography demonstrated soft tissue density in their paranasal cavities. With the exception of CRS, the participants were healthy. None had a history of renal, liver, or blood disease, nor were they taking any other medications that might have affected metabolism and distribution of bepotastine besilate. Among them, 10 participants had allergic rhinitis (AR) (Group I), and others did not have AR (Group II). AR in the participants of Group I had been diagnosed as persistent allergic rhinitis by their clinical symptoms, plus signs, and serum-specific Immunoglobulin E (IgE) based on the ARIA's definition [3] (Table 1).

Nasal mucosa was collected from the anterior portion of the inferior turbinate using forceps under general anesthesia 55 to 130 minutes after single-dose oral administration of bepotastine besilate 10 mg. There was no significant difference in the sampling times between the two groups (*t*-test, *P* = .1178). The mean weight of the nasal mucosa was approximately 24 mg. Blood was collected at the same time as the collection of the nasal mucosa. The blood was immediately centrifuged to obtain the serum. The serum and nasal mucosa were promptly frozen and preserved at −40°. Concentrations of the agent in the serum and tissue were measured by high-performance liquid chromatography using an API-4000 LC-MS-MS system (Applied Biosystems Co., Ltd, California, USA). The sensitivity and reproducibility of the LC-MS-MS system were 98.0% and 98.9%, respectively. Chromatographic separation was achieved on a Capcell Pak MG II C18 (2.0 × 150 mm, 3 *μ*m; Shiseido, Tokyo, Japan), and the column temperature was maintained at 40 degrees. The lower limit of quantitation for serum and nasal mucosa was 1 ng/mL and 2 ng/g, respectively.

To compare the concentration of bepotastine besilate of the serum and nasal mucosa tissue in each group, we statistically evaluated the data with a paired *t*-test. To compare the concentration of bepotastine besilate in the nasal mucosa tissue between the two groups, we used Mann-Whitney's U-test.

The protocol was approved by our hospital's ethics committee and informed consent was obtained from all the participants. 

## 3. Results

The concentration of bepotastine besilate of the serum in Groups I and II ranged from 67.98 to 155.98 ng/mL (a mean and standard deviation: 98 ± 32) and from 65.04 to 197.55 ng/mL (112 ± 39) (Table 1). Those of the nasal mucosa tissue in Groups I and II ranged from 47.79 to 186.1 ng/g (101 ± 36) and from 57.86 to 187.01 ng/g (132 ± 44) (Table 1).

**Figure 1:**
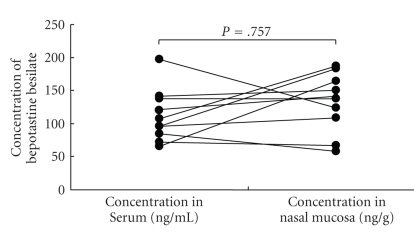
The graph showed the result of the concentration of bepotastine besilate in the serum and nasal mucosa of chronic rhinosinusitis with allergic rhinitis and compares using a paired *t*-test.

**Figure 2:**
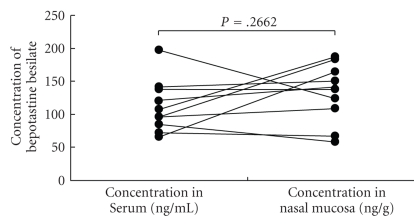
The graphs demonstrated the result of the concentration of bepotastine besilate in the serum and nasal mucosa of chronic rhinosinusitis without allergic rhinitis and compares using a paired *t*-test.

**Figure 3:**
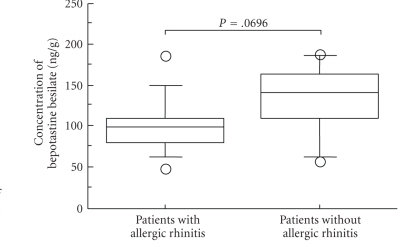
The box plot graph showed the result of the concentration of bepotastine besilate in the nasal mucosa of chronic rhinosinusitis with and without allergic rhinitis and compares using Mann-Whitney's U-test.

There was no significant difference in the values of concentration of bepotastine besilate between the serum and the nasal mucosa in either Group I or II (*P* = .757 and *P* = .2662, Paired *t*-test, Figures 1 and 2). There was a tendency for the concentration of bepotastine besilate in the nasal mucosa to be higher in Group II than in Group I (*P* = .0696, Mann-Whitney's U-test) (Figure 3). These results may indicate swift absorption and transition of bepotastine besilate from the gastrointestinal tract to blood and from blood to tissue. 

## 4. Discussion

P-glycoproteins, which affect the tissue concentration of the drugs, were reported to be expressed in the human nasal mucosa [4]; hence we guess whether the tissue concentration of the drug is the same as the serum concentration of it or not. In fact, there were many reports on the tissue concentration of antimicrobial and anticancer drugs in the human nasal and paranasal region [5–7]. Histamine is an important chemical mediator in allergic rhinitis and plays an important role in eliciting the nasal symptoms of the disorder [8]. H1-receptors are also known to exist on the postcapillary venules in nasal mucosa to induce vasodilation, to increase vascular permeability, and to stimulate sensory nerve endings in response to histamine [9]. There has been a lot of literature reporting the concentration of H1-receptor antagonists in the serum [1, 2, 10] and there was only one report about it in human skin [11]. However, to our knowledge, there has been no report actually measuring the concentration of H1-receptor antagonists in nasal mucosa. Therefore, this preliminary study is considered the first report of its kind. 

It is known that the rise of the concentration of bepotastine besilate in the serum after administration is more rapid than the other H1-receptor antagonists and that the *t*-max and half-lives (*t*1/2) in the serum were 1.2 hours and 2.4 hours, respectively [2]. Therefore, we collected the blood and nasal mucosa less than 2.4 hours after single-dose oral administration of bepotastine besilate. There was no significant difference in the values of concentration of bepotastine besilate between the serum and the nasal mucosa. These results may indicate swift absorption and transition of bepotastine besilate from the gastrointestinal tract to blood and from blood to tissue. The results of the present study indicating prompt absorption and transition of bepotastine besilate seem to support the favorable effects of bepotastine besilate, which was reported to reveal a faster clinical effect of bepotastine besilate [12]. 

In the present study, we chose the CRS patients because biopsy specimens could easily be taken. We feared that the pathophysiologic changes accompanying CRS would not make any statement regarding the metabolism and tissue distribution of the bepotastine besilate. However, the values of concentration of bepotastine besilate between the serum and the nasal mucosa were almost the same. Therefore, we think that pathophysiologic changes are small in the inferior turbinate accompanying CRS without AR. However, we surmise that allergic conditions (edema, etc.) may cause pathophysiologic changes in the nasal mucosa, because the concentration of bepotastine besilate in the nasal mucosa was lower in allergic conditions than in nonallergic conditions.

The values of concentration of bepotastine besilate both in the serum and nasal mucosa were found to vary greatly. Variations may result from variation of sampling time, individual variation of absorption, metabolism and excretion of bepotastine besilate, and/or individual variation of its pharmacokinetics in nasal mucosa. Also, it is necessary for pharmacokinetics in the nasal mucosa to be sampled at least two times.

## Figures and Tables

**Table 1:** Characters of the participants and results of the concentration of BB in the serum and nasal mucosa.

				Sampling time after	Concentration	Concentration	
	Case	Age (years)	Sex	oral administration	in the serum	in the nasal mucosa	Antigen
				of BB (h:m)	(ng/mL)	(ng/g)	
Group I	1	53	Male	1:10	155.98	111.05	Amb, Asp, Candida, Cha, Cry, Dp, HD
	2	25	Female	1:30	132.34	108.3	Cha, Cry, Dp, HD
	3	29	Male	1:35	67.98	47.79	Cha, Cry, Dp, HD
	4	68	Male	1:40	98.14	76	Dp, HD
	5	68	Male	1:40	74.62	79.57	Candida, Dp, HD
	6	31	Male	1:45	49.35	97.3	Cha, Cry, Dp, HD
	7	60	Male	1:50	86.85	99.2	Candida, Cha, Cry, Dp, HD
	8	55	Male	2:00	109.27	108.6	HD
	9	17	Female	2:00	83.5	98	Amb, Asp, Candida, Dp, HD
	10	13	Male	2:10	119.41	186.1	Cry, Dp
Group II	11	38	Female	0:55	96.22	183.67	non
	12	57	Male	1:00	71.38	66.7	non
	13	55	Female	1:20	137.21	138.2	non
	14	52	Male	1:20	121.58	141.5	non
	15	46	Male	1:30	65.04	163.88	non
	16	64	Female	1:30	107.48	187.01	non
	17	52	Female	1:40	197.55	123.85	non
	18	50	Male	1:40	85.14	57.86	non
	19	76	Male	2:00	141.6	150.57	non
	20	56	Male	2:00	95.47	108.7	non

Amb: Ambrosia elatior; Asp: Aspergillus; BB: Bepotastine Besilate; Cha: Chamaecyparis obtuse; Cry: Cryptomeria japonica; Dp: Dermatophagoides pteronyssinus; HD: Housedust.
